# Age-Friendly Cities and Communities: An International Perspective

**DOI:** 10.1093/geroni/igaf122.1195

**Published:** 2025-12-31

**Authors:** Kathrin Boerner, Nina Silverstein

## Abstract

Given population aging and urbanization, cities and communities must evolve meeting the needs and foster the potential of older adults. The Age-Friendly Cities framework, launched by the World Health Organization in 2007, provides a structured approach to assessing and enhancing urban environments for aging populations. It specifies eight interconnected domains of urban life: Outdoor Spaces and Buildings, Transportation, Housing, Social Participation, Respect and Social Inclusion, Civic Participation and Employment, Communication and Information, Community Support and Health Services. This symposium brings together international research on developing and implementing age-friendly initiatives, offering insights from different countries. Grenz and colleagues present a study using the Age-Friendly Cities and Communities Questionnaire (AFCCQ) in Germany, identifying four distinct subgroups of older adults based on socio-demographic profiles and evaluations of age-friendliness. Marston and colleagues assess the AFCCQ in the UK, demonstrating its intergenerational applicability and revealing financial disparities as a key factor in age-friendliness perceptions. Ayalon and colleagues examine the AFCCQ in Israel, highlighting variations in satisfaction across cities, with social inclusion and respect emerging as critical concerns. Siebecker and Coyle present findings from the second cycle of Age-Friendly Boston, illustrating how older adults’ priorities have shifted over time from housing and economic security to social connection and civic engagement. By considering findings across different national and cultural contexts, this symposium provides a cross-cultural view and comparison of age-friendly city initiatives. The discussion will emphasize the importance of data-driven urban planning, targeted interventions, and international collaboration in creating inclusive and sustainable environments for aging populations worldwide.

